# Supplementary material for: Cloud BioLinux: pre-configured and on-demand bioinformatics computing for the genomics community
Source: BMC Bioinformatics. 2012 Mar 19;13:42. doi: 10.1186/1471-2105-13-42 (PMC3372431; doi:10.1186/1471-2105-13-42)
Supplement: Additional file 1 — Supplementary 1 Cloud BioLinux software documentation in the form of a mini, self-contained website. Users need to download and uncompress the .zip file, and open through a web browser the "index.html" file available on the main directory. (ZIP 1823 kb). [file 1471-2105-13-42-S1.ZIP › Cloud-BioLinux-Package-Documentation/list_app.html]

Bio-Linux Software Documentation Pages

Back to search form

### Browse by Application/ Package Name

|  |  |  |  |  |  |  |  |  |
| --- | --- | --- | --- | --- | --- | --- | --- | --- |
| A; aaindexextract abiview acdc acdlog acdpretty acdtable acdtrace acdvalid act afetch ajbad ajfeatest ajtest aligncopy aligncopypair alistat annotate anomaly antigenic ape arb artemis assembly-conversion-tools atv | B; backtranambig backtranseq banana baseml basemlg big-blast bioconductor BiodiversityR biojava bioperl bioruby biosed bl2seq blast+ blast2 blastall blastcl3 blastclust blastdbcmd blastn blastp blastpgp blastx blixem boxshade BTmapqtl btwisted build-icm bwa | C; caf2gap caftools cai cap3 cd-hit cd-hit-est cdbfasta cdbyank chaos charge check checktrans chi2 chimeraslayer chips cirdna clcsequenceviewer clique clmclose clmconf clmdist clmformat clmimac clminfo clmmate clmmeet clmresidue clobb clustalw clustalx cn3d codcmp codcopy codeml coderet codon-usage combineMUMs compare-lists complex compseq cons consambig consed consense contml contrast copymat corbatest cpgplot cpgreport cross\_match cusp cutgextract cutseq cytoscape | D; dan dba dbiblast dbifasta dbiflat dbigcg dbxfasta dbxflat dbxgcg dbxstat degapseq demoalign demofeatures demolist demostring demostringnew demotable dendroscope denoiser density descseq dialign diffseq digest distmat dnacomp dnadist dnainvar dnaml dnamlk dnamove dnapars dnapenny dollop dolmove dolpenny dotmatcher dotpath dotter dottup dotur drawgram drawtree dreg dust dustmasker | E; edialign einverted Emap embossdata embossversion emma emowse entrails entret Entrez epestfind eprimer3 Eqtl equicktandem est2genome estscan estwise estwisedb etandem evolver exact-tandems extract extractalign extractfeat extractseq extract\_from\_pdb | F; factor fasta fastacmd fastdnaml fastf fastm FastQC fasts FastTree fastx fasty featcopy featreport findkm fitch flalign formatdb formatrpsdb freak fuzznuc fuzzpro fuzztran | G; gap2caf gap4 gaps garlic garnier gcphrap geecee gendist generate genespring genewise genewisedb get-len get-putative getorf gff2ps ggsearch giep glam2 glimmer2 glsearch gromacs gtop | H; HAPPY helixturnhelix histogramtest hmmalign hmmbuild hmmcalibrate hmmcalibrate-pvm hmmconvert hmmemit Hmmer hmmfetch hmmindex hmmpfam hmmpfam-pvm hmmsearch hmmsearch-pvm hmoment htop | I; iep impala infoalign infobase inforesidue infoseq intconv isochore |
 J; jalview jaspextract jaspscan jMOTU jprofilegrid JZmapqtl | K; kitsch | L; lalign lalign2list Lamarc lav2ps lav2svg lfasta lindna listor long-orfs LRmapqtl lucy | M; makeblastdb makemat makembindex makenucseq makeprotseq mapview maq marscan maskambignuc maskambigprot maskfeat maskseq matcher maxdLoad2 maxdview mcl mclfamily mclfaq mclpipeline mcmctree mcx mcxarray mcxassemble mcxconvert mcxdump mcxmap mcxsubs mdust megablast megamerger merger mesquite mgaps migrate MImapqtl mira mira-3rd-party mix mocca mothur move mrbayes mrbayes-multi msbar mspcrunch MultiRegress mummer mummerplot muscle mview mwcontam mwfilter | N; needle needleall neighbor netblast newcoils newcpgreport newcpgseek newseq njplot nohtml noreturn nospace notab notseq nrdb nthseq nthseqset nucmer nucmer2xfig | O; ocount octanol oddcomp OligoArray omssa origsplitter origunion | P; palindrome pamp pars partigene pasteseq patmatdb patmatmotifs patmattest pedro penny pepcoil pepinfo pepnet pepstats peptidemapper pepwheel pepwindow pepwindowall pfmake pfscale pfscan pfsearch pftools pfw phrap phred phylip Phyml plalign plfasta plotcon plotorf polydot postnuc postpro prank preg pregap4 prenuc Preplot prepro prettyplot prettyseq prfx priam prima primer3 primers primersearch printsextract profit PROmer proml PROmlk prophecy prophet prosextract prot4est protdist protpars prss Prune psa2msa pscan psiblast psiphi psw pswdb ptof ptoh | Q; qiime Qstats QTLcart QTL\_Cartographer | R; R rasmol Rcmdr Rcross rdp\_classifier readseq rebaseextract recoder redata remap repeat-match restdist restml restover restrict retree revseq Rmap roche2gap rpsblast rpsblast+ rpstblastn Rqtl run-glimmer2 run-mummer1 run-mummer3 | S; samtools seaview seealso seedtop segmasker seqboot seqinfo seqmatchall seqret seqretall seqretallfeat seqretset seqretsetall seqretsingle seqretsplit seqrettype seqsplit seqstat sequin sfetch show-aligns show-coords show-tiling showalign showdb showfeat showorf showpep showseq shuffle shuffleseq sibsim4 sigcleave silent sim4 sindex sirna sixpack sizeseq skipredundant skipseq spin splitsource splitstree splitter squint sreformat SRmapqtl ssake ssearch stars stretcher stssearch supermatcher swat syco | T; t-coffee Taverna taxblast tblastn tblastx tcode testplot textsearch tfastf tfastm tfasts tfastx tfasty tfextract tfm tfscan tmap tqs trace2dbest tranalign transeq tree-puzzle treedist treeview trev trimest trimseq trimspace twofeat | U; uclust union | V; vectorstrip velvet | W; water weight whichdb windowmasker wobble wordcount wordfinder wordmatch wossname | X; xsplits | Y; yamap yank yn00 | Z; Zmapqtl |
